# ITGB4 deficiency induces mucus hypersecretion by upregulating MUC5AC in RSV-infected airway epithelial cells

**DOI:** 10.7150/ijbs.66215

**Published:** 2022-01-01

**Authors:** Xizi Du, Yu Yang, Ming Yang, Lin Yuan, Leyuan Wang, Mengping Wu, Kai Zhou, Wenkai Li, Yang Xiang, Xiangping Qu, Huijun Liu, Xiaoqun Qin, Chi Liu

**Affiliations:** 1 Department of Physiology, School of Basic Medicine Science, Central South University, Changsha, Hunan, China; 2 Centre for Asthma and Respiratory Disease, School of Biomedical Sciences and Pharmacy, Faculty of Health and Medicine, University of Newcastle and Hunter Medical Research Institute, Callaghan, New South Wales, Australia; 3 Department of Pediatrics, Hunan Provincial People's Hospital, The First Affiliated Hospital of Hunan Normal University, Changsha, Hunan, China; 4 Research Center of China-Africa Infectious Diseases, Xiangya School of Medicine Central South University, Changsha, Hunan, China

**Keywords:** respiratory syncytial virus, MUC5AC, ITGB4, airway epithelial cell

## Abstract

Respiratory syncytial virus (RSV) infection is the main cause of bronchiolitis in children. Excessive mucus secretion is one of the primary symbols in RSV related lower respiratory tract infections (RSV-related LRTI), which is closely associated with the occurrence and development of asthma in later life. Integrin β4 (ITGB4) is down-regulated in the airway epithelial cells (AECs) of asthma patients which plays a critical role in the pathogenesis of asthma. However, whether ITGB4 is involved in the pathological processes of RSV infection remains unclear. In this study, we found that decreased expression of ITGB4 was negatively correlated with the level of MUC5AC in childhood AECs following RSV infection. Moreover, ITGB4 deficiency led to mucus hypersecretion and MUC5AC overexpression in the small airway of RSV-infected mice. MUC5AC expression was upregulated by ITGB4 in HBE cells through EGFR, ERK and c-Jun pathways. EGFR inhibitors treatment inhibited mucus hypersecretion and MUC5AC overexpression in ITGB4-deficient mice after RSV infection. Together, these results demonstrated that epithelial ITGB4 deficiency induces mucus hypersecretion by upregulating the expression of MUC5AC through EGFR/ERK/c-Jun pathway, which further associated with RSV-related LRTI.

## Introduction

Respiratory syncytial virus (RSV) is one of the most common respiratory pathogens causing lower respiratory tract infection among neonates and infants. RSV infection results about 33 million acute lower respiratory tract infections (LRTI) cases worldwide each year. Not only that, RSV-related LRTI would induce subsequent severe respiratory diseases and around 55,000-199,000 deaths in children younger than the age of 5 years old, which poses a heavy world medicine burden 1.

Airway epithelial cells (AECs), specifically ciliated AECs, is the main target for RSV infection. Of particular concern is that mucus hypersecretion in AECs is one of most important pathological events in severe LRTI, which is always associated with increased risk of subsequently wheezing and later asthma in childhood 2, 3. Mucus is important for the mucociliary clearance system, which is the first line against exogenous stimuli and pathogens 4. However, mucus hypersecretion in pathological state would aggravate inflammatory response, which further led to airway hyperresponsiveness (AHR), airflow obstruction and weakened lung function 5. Thus, exploring the intrinsic mechanism that causes abnormal mucus secretion in RSV-infected AECs will contribute to prevent and reduce the clinically occurrence of severe LRTI.

Mucins, the main components of mucus, have many different types. In addition to forming a protective barrier against pathogens, mucins also have other unconventional functions, such as anti-inflammatory action and receptors for bacterium 6. MUC5AC and MUC5B are the principal secretory mucins in the airway and MUC5AC is the primary gene expressed by airway epithelial goblet cells. Generally, MUC5AC is mainly expressed at relatively low level. However, the expression of MUC5AC increased significantly in inflammatory microenvironment which was usually caused by various external stimuli including RSV infection 6, 7. Studies have found that MUC5AC overexpression induced the hypersecretion of airway mucus in RSV-infected AECs 8, 9. However, the mechanisms underlying MUC5AC overexpression in RSV-infected AECs remain largely unclear.

Integrin family is a type of adhesion molecules that are constitutively expressed in AECs. Among these molecules, integrin β4 (ITGB4) deserves a special attention for its unique intracellular long subunit. ITGB4 has been proven to act a critical role in the activation of phosphatidylinositol 3-kinase (PI3K), extracellular signal-regulated kinases (ERK1/2), nuclear factor kappa-B (NF-κB) and other intracellular signaling pathways 10-12. Of note, expression of ITGB4 decreased significantly by specific variation sites in the AECs of asthma patients 13. Moreover, ITGB4 deficiency induces exaggerated lung inflammation and enhanced AHR in House Dust Mite (HDM) treated mice 14.

Although the association between ITGB4 and asthma has been verified, little is known about the contribution of ITGB4 to mucus hypersecretion after RSV infection in childhood asthma. Thus, we further though to unravel the role of ITGB4 in the regulation of mucus production following RSV infection in AECs. First, RSV-infected model was constructed both *in vitro* and *in vivo* to investigate mucus production in ITGB4 deficient AECs following RSV infection. Then, throat swab samples from children's cases of RSV induced bronchiolitis and control cases were collected to verify the correlation between ITGB4 and MUC5AC in RSV-infected airway epithelia. Finally, corresponding intervention strategies were performed to identify the underpinning molecular mechanisms of MUC5AC overexpression in ITGB4 deficient AECs after RSV infection.

## Material and methods

### Throat swab sample collection

All participants provided written informed consent, approved by the No.2020KT-52 Central South University Research Ethics Committee. Children cases of RSV infection and healthy controls were recruited at Hunan Provincial People's Hospital between September 2020 and February 2021. Eligible patients (RSV patients' group) were aged under 18 and diagnosed with bronchiolitis and RSV-RNA positive. Patients who had pneumonia, acute heart failure within a month were excluded. Healthy controls were aged under 18 without respiratory disease. The demographic and clinical characteristics are shown in Table S2. Throat swab samples were collected for extracting RNAs and qPCR analysis by using BIOG RNA Swab Kit (BAIDAI, China).

### Animals and challenge protocols

This animal studies were approved by the Xiangya Animal Protection and Use Committee of Central South University (No. 2020sydw0305). ITGB4 conditionally knocked out mice (ITGB4^-/-^ mice) was constructed according to our previous work 15. The AECs-specific ITGB4 knock out was controlled by doxycycline (Dox) induction. After birth, Dox was placed in drinking water (1mg/ml) for mother. Infant mice are breastfed and injected with Dox (i.p. 25mg/kg) on day 7 and 14 after birth. ITGB4^fl/fl^ male littermates lacking either CCSP-rtTA or TetO-Cre were served as controls (ITGB4^+/+^ mice) which were treated with Dox synchronously. For the induction of RSV infection, mice were intranasally (i.n) stimulated with 1×10^6^ pfu RSV-A2 in 20 ul on day 15 after birth, and the control group received an equal volume of PBS 16. Lung tissues were collected on day 17, 19, and 21 after birth (mice killed by exsanguination following an overdose of sodium pentobarbital). All the animal work was taken place in Department of Laboratory Animals, Central South University.

### PAS staining

The PAS staining was performed following Glycogen Periodic Acid Schiff (PAS/Hematoxylin) Stain Kit (Solarbio, China) protocol 17.

### Cell culture, transfection of siRNA, RSV infection, inhibitors treatment

Human bronchial epithelial (HBE) cells were purchased from Lifeline Cell Technology (Frederick, MD, USA), HBE cells were cultivated in DMEM with 10%FBS, 50U/ml Penicillin, 50U/ml Streptomycin. ITGB4 siRNA (50nM) or negative control siRNA (NC siRNA, 50nM) (RiboBio Inc, Guangzhou, China) were transfected into HBE cells with LipofectAMINE 3000 (Invitrogen, Carlsbad, CA, USA). For the induction of RSV stimulation, HBE cells were incubated with RSV-A2 for 24 hours. In the group of inhibitor treatment, cells were pretreated for 30 min with AG1478 (1 μM, EGFR phosphorylation inhibitor), U0126 (10 μM, ERK phosphorylation inhibitor) or SP600125 (5μM, JNK inhibitor) (Selleck Chemicals, USA) respectively before incubation with RSV-A2.

### RNA extraction, RT-PCR and qPCR

Total RNA was extracted by Trizol and quantified on a Varioskan microplate reader (Thermo Scientific, USA). PCR and qPCR were conducted according to the Kit protocol (PrimeScript RT Master Mix Kit, Takara, Japan) 15. Primer sequences were shown in Table S1.

### Western blot analysis

Protein was extracted from HBE cells according to previous procedures 18. The following antibodies were used to determine the expression of corresponding protein: ITGB4 (ab197772, Abcam, USA), EGFR (ab52894, Abcam, USA), p-EGFR (ab32430, Abcam, USA), ERK1/2(ab184699, Abcam, USA), p-ERK1/2(ab223500, Abcam, USA), c-Jun (sc-74543, Santa Cruz, USA), p-c-Jun (sc-822, Santa Cruz, USA). Lamin-β1(ab133741, Abcam, USA) and β-actin (ab8226, Abcam, USA) were used as corresponding controls, respectively.

### Immunoprecipitation

Immunoprecipitation was performed according to our previous publications 19. Briefly, protein from HBE cells was extracted with weak RIPA lysis buffer. An appropriate dilution of anti-ITGB4 antibody (Ab197772, Abcam, USA) or anti-EGFR antibody (sc-373746, Santa Cruz, USA) were added to the centrifuge tube which conjugate with suspension of Protein G. Mix the antibody-bead mixture at 4°C for 4 h using tube rotator. Then, 50μg of cell lysates were added into the mixtures and the lysate-bead/antibody conjugate mixtures were incubated overnight at 4°C. Mixtures were further resuspended in 5xSDS loading buffer. Samples were boiled for 5 minutes and analyzed by Western blot. Immunocomplexes were stained with anti-ITGB4 (ab197772, Abcam, USA) as well as EGFR (sc-373746, Santa Cruz, USA).

### Immunocytochemical staining

HBE cells slides were fixed with 4% paraformaldehyde and incubated with PBS (containing 0.1% Triton X-100) for 10 minutes. After blocking with 1% BSA, cells were incubated with antibodies for MUC5AC (sc-21701, Santa Cruz, USA) or c-Jun (sc-74543, Santa Cruz) in a humidified box and incubate overnight at 4°C. Then, cells were incubated with DAPI for DNA staining (Solarbio, China). Results were visualized using a fluorescence microscope (Carl Zeiss MicroImaging GmbH, Göttingen, Germany).

### Statistical analysis

Pearson' correlation was used to access the association between the expression of ITGB4 and MUC5AC, which was done with SPSS.19. Other data were analyzed with GraphPad Prism Software (Version 7; San Diego, CA) and presented as mean ± SEM. Statistical comparisons were made by one-way ANOVA followed by Dunnett's post hoc test. Differences were considered statistically significant for *p < 0.05, **p < 0.01 and ***p < 0.001.

## Results

### ITGB4 deficiency in AECs induced mucus hypersecretion in RSV-infected mice

**Figure 1:**
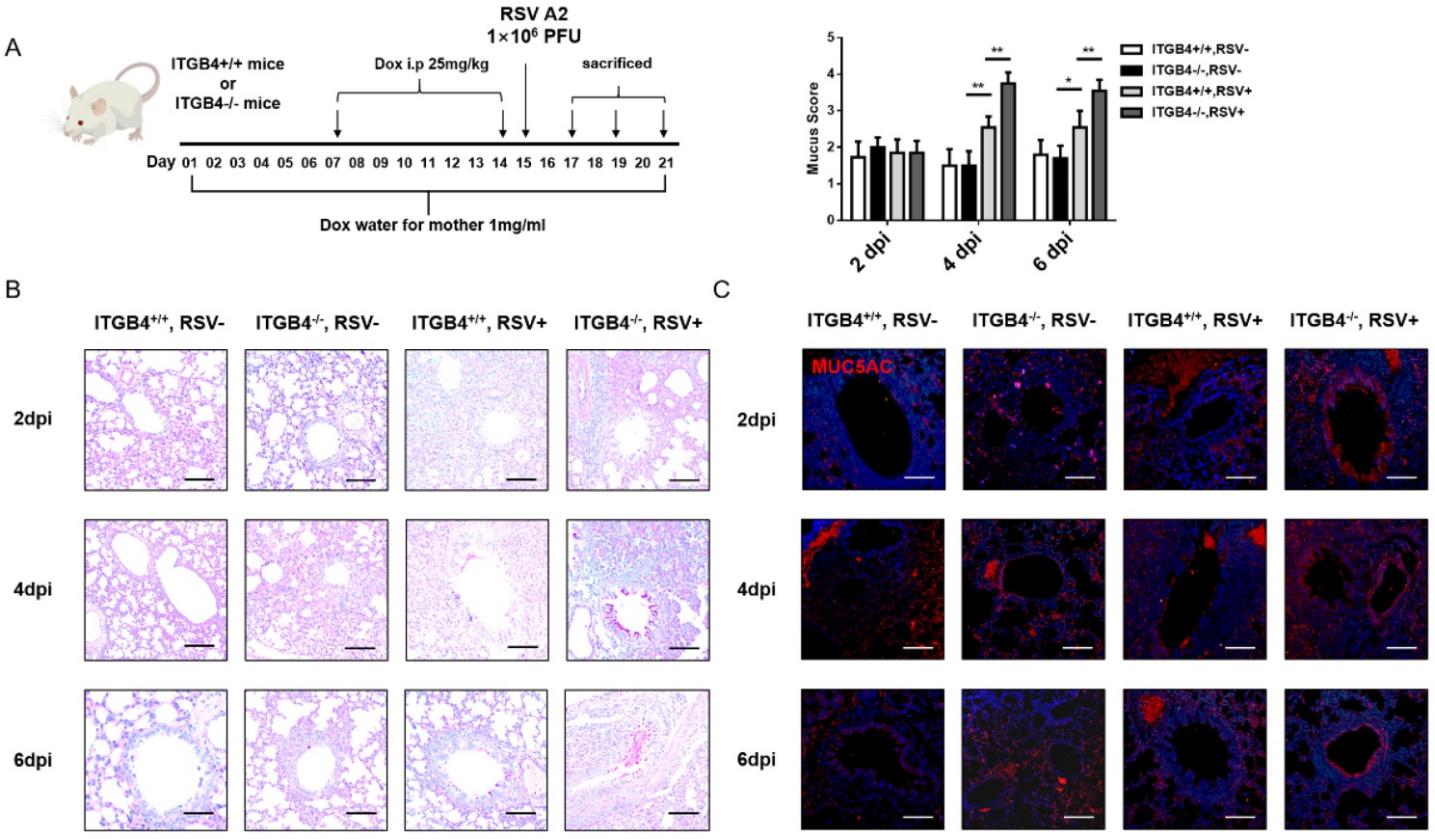
ITGB4 deficiency contributes to mucus hypersecretion and MUC5AC overexpression after RSV infection. A. Dox was placed in drinking water for mother and passed through breast milk to infant mice. Infant mice were injected Dox (i.p) on day 7 and 14 after birth. RSV was stimulated on day 15. Control mice received PBS. B. Mucus secretion were assessed by PAS staining. C. The level of MUC5AC were assessed by immunofluorescence (n = 10). Scale bar, 25 μm. * p<0.1; **p<0.05.

To explore the influence of ITGB4 deficiency on the mucus secretion in airway epithelia following RSV infection, RSV-infected mice animal was constructed (Figure 1A). PAS staining showed that, on day 2 post RSV infection, a small amount of mucus was detected in bronchial lumen of both ITGB4^-/-^ mice and ITGB4^+/+^ mice. On day 4 and day 6 post infection, goblet cells in the bronchial lumen of ITGB4^-/-^ mice were significantly proliferated and mucus secretion was greatly increased, whereas ITGB4^+/+^ mice showed no obvious goblet cell proliferation and mucus secretion (Figure 1B). Meanwhile, during days 4 to 6 post RSV infection, the expression of MUC5AC in ITGB4^-/-^ mice were significantly higher than that in ITGB4^+/+^ mice (Figure 1C, Figure S1).

### Involvement of ITGB4 deficiency on the overexpression of MUC5AC in RSV patients

**Figure 2:**
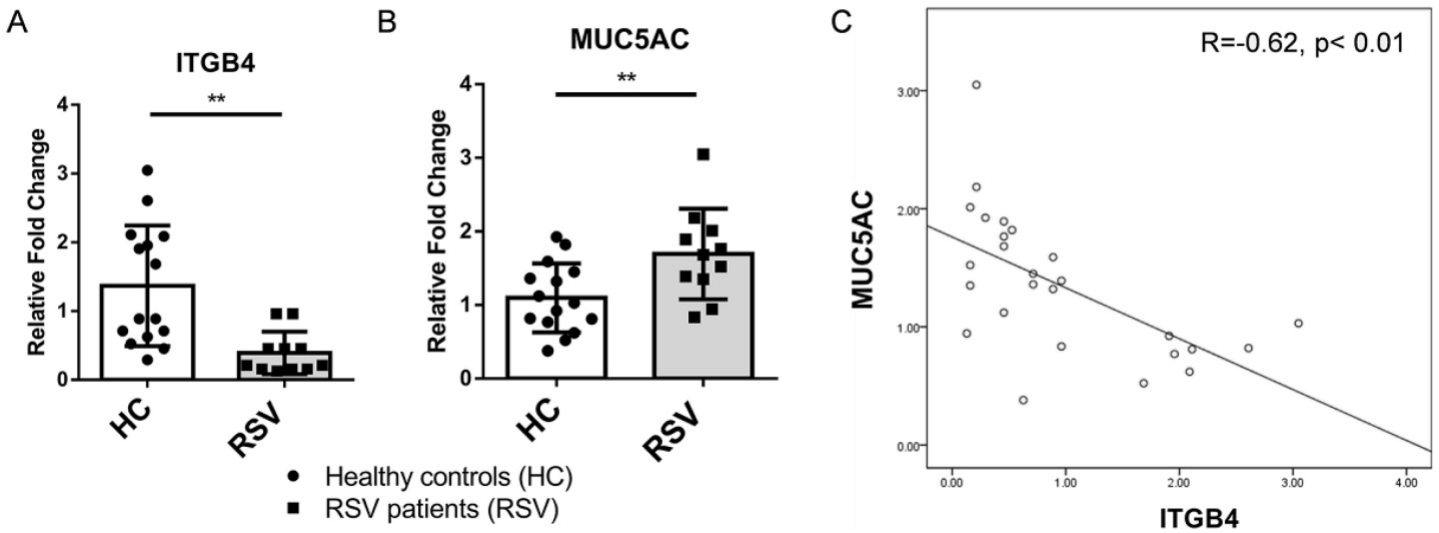
Expression of ITGB4 were negatively associated with level of MUC5AC in RSV patients. A. mRNA expression of ITGB4 in throat swab samples from RSV patients (n=11) and healthy controls (n=15). B. mRNA expression of MUC5AC in throat swab samples from RSV patients and healthy controls. C. Correlation analysis between ITGB4 expression and level of MUC5AC in RSV patients. **p<0.05.

Both clinic and animal studies have shown that mucus hypersecretion from airway epithelia is one of most important pathological events in severe LRTI following RSV infection. To further determine the impact of ITGB4 on mucus hypersecretion, we examined the levels of MUC5AC through throat swab samples from RSV patients and healthy controls. Compared with healthy controls, expression of ITGB4 decreased significantly in RSV patients, (Figure 2A), while levels of MUC5AC were higher in RSV patients compared with healthy controls (Figure 2B). Of note, expression of ITGB4 was negatively associated with the level of MUC5AC in RSV patients (Figure 2C). This correlation strongly indicated that ITGB4 deficiency in airway epithelia was involved in mucus hypersecretion and MUC5AC overexpression following RSV infection.

### Level of MUC5AC was induced significantly in ITGB4 deficient HBE cells after RSV infection

**Figure 3:**
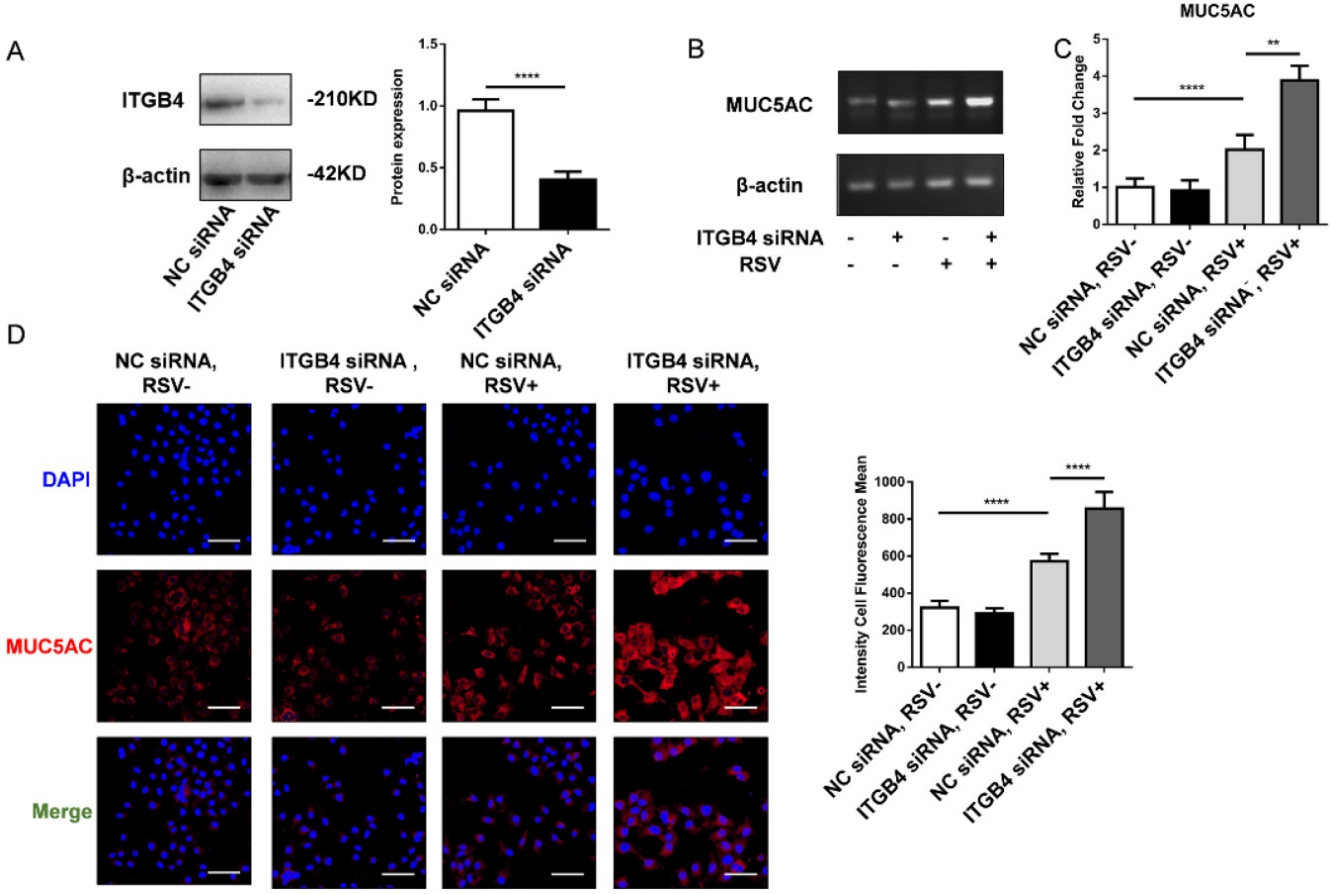
The level of MUC5AC increased significantly in ITGB4 silenced HBE cells after RSV infection. A. The silence efficiency of ITGB4 was verified by western blot. B-D. ITGB4 deficiency significantly upregulated the mRNA and protein level of MUC5AC, respectively. Immunofluorescence were quantified by the fluorescence intensity (n = 10). Scale bar, 50 μm. Values are represented as mean ± SEM of at least three independent experiments. ** p<0.05; **** p<0.001.

To further verify the effect of mucus hypersecretion caused by ITGB4 deficiency, HBE cells were transfected with ITGB4 siRNA, respectively. The silence efficiency of ITGB4 was verified by western blot (Figure 3A). After RSV infection, the mRNA expression of MUC5AC enhanced significantly in ITGB4 silenced HBE cells. Besides, comparing with control HBE cells, the protein expression of MUC5AC in ITGB4 silenced HBE cells also increased obviously following RSV infection (Figure 3B, C, D).

### ITGB4 deficiency increased EGFR phosphorylation in RSV-infected HBE cells

**Figure 4:**
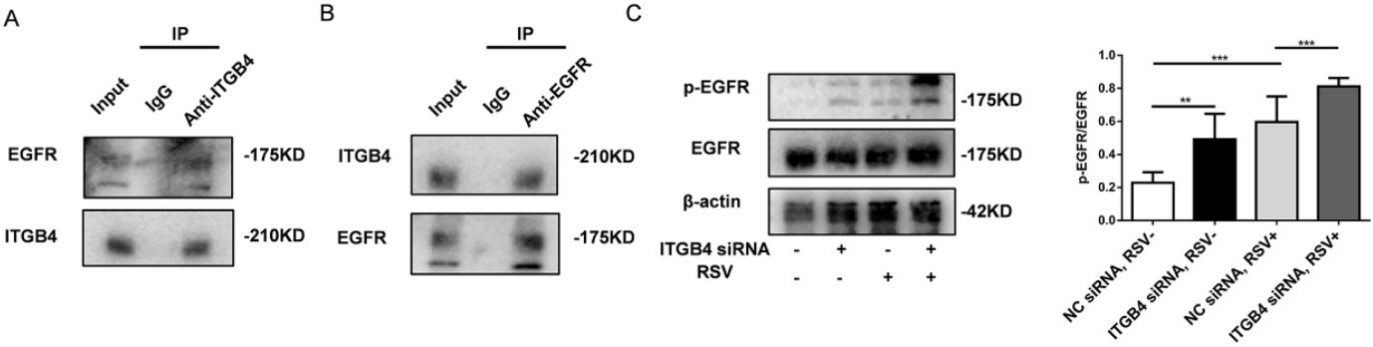
The interaction between ITGB4 and EGFR and the phosphorylation of EGFR in ITGB4 silenced HBE cells after RSV infection. A-B. Immunoprecipitation of RSV-infected HBE cells for interaction between ITGB4 and EGFR. C. ITGB4 deficiency enhanced EGFR phosphorylation in RSV-infected HBE cells, as compared with that in control HBE cells. Values are represented as mean ± SEM of at least three independent experiments. ** p<0.05; *** p<0.01.

Previous literatures have showed that ITGB4 reduces the binding of ligand to EGFR and the mobility of EGFR in the plasma membrane through physical interaction with EGFR 20-22. In this study, we further determined whether ITGB4 regulates EGFR phosphorylation in RSV-infected HBE cells. Assay with immunoprecipitation revealed a direct interaction between ITGB4 and EGFR in RSV-infected HBE cells (Fig 4A, B). Basal level of EGFR phosphorylation in RSV-infected HBE cells was higher than that in control HBE cells without outside stimuli. Moreover, after RSV infection, ITGB4 silence further enhanced EGFR phosphorylation in HBE cells compared with that in control HBE cells (Fig 4C). Either at baseline or after RSV infection, there is no significant difference of total EGFR between ITGB4 silenced HBE cells and control HBE cells. The above results indicated that ITGB4 was involved in the activation of EGFR during RSV infection.

### ITGB4 deficiency enhanced the activation of EGFR/ERK/c-Jun pathway in RSV-infected HBE cells

**Figure 5:**
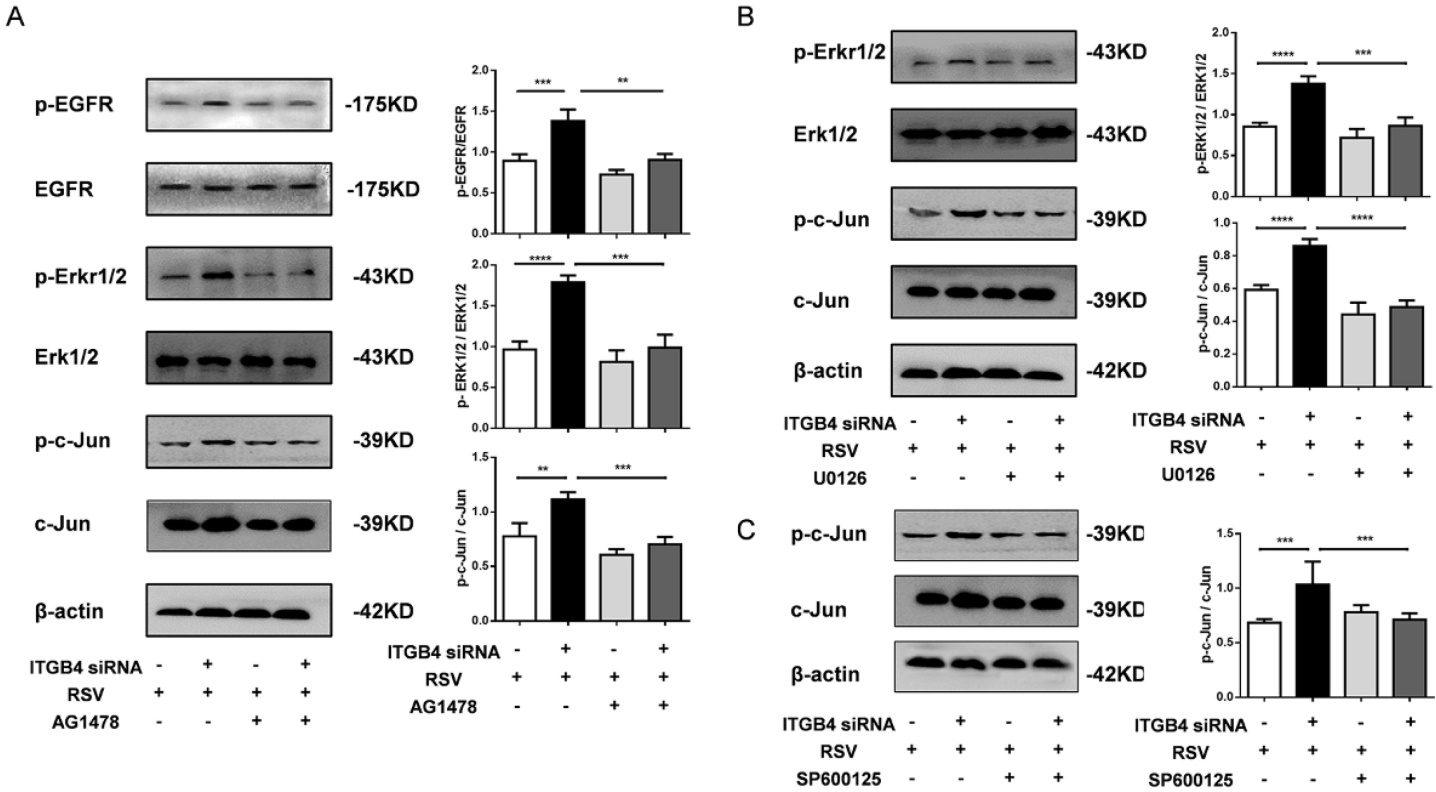
ITGB4 deficiency leads to augmented activation of EGFR/ERK1/2/c-Jun pathway. A. AECs from different group was pretreated with AG1478 and then infected with RSV. Western blot analysis of the expressions of p-EGFR, EGFR, p-ERK1/2, ERK1/2, p-c-Jun, c-Jun. B. HBE cells from different group was pretreated with U0126 and then infected with RSV. Western blot analysis of the expressions of p-ERK1/2, ERK1/2, p-c-Jun, c-Jun; C. HBE cells from different group was pretreated with SP600125 and then infected with RSV. Western blot analysis of the expressions of p-c-Jun and c-Jun; Values are represented as mean ± SEM of at least three independent experiments. ** p<0.05; *** p<0.01; **** p<0.001.

To further explore the role of EGFR mediated signaling pathways in ITGB4 deficiency-induced MUC5AC overexpression, we examined the downstream pathway of EGFR activation in ITGB4 silenced HBE cells after RSV infection. The expression of ERK1/2, phosphorylated ERK1/2 (p-ERK1/2), c-Jun and phosphorylated c-Jun (p-c-Jun) were detected in ITGB4 silenced HBE cells after RSV infection using multiple inhibitors, including AG1478, U0126 and SP600125. Western blot analysis showed that treatment with AG1478, U0126 and SP600125 significantly inhibited the level of p-EGFR, p-ERK1/2 and p-c-Jun in ITGB4 silenced HBE cells after RSV infection, respectively (Figure 5A, B, C).

**Figure 6:**
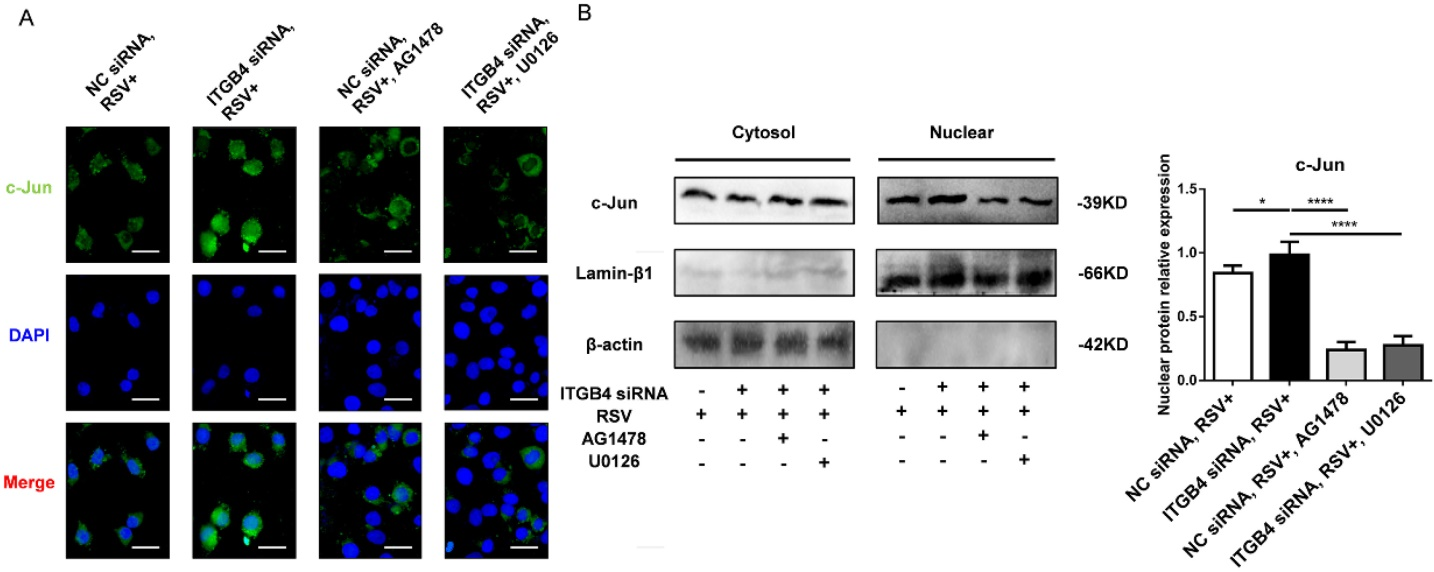
Nuclear extract for c-Jun was detected in ITGB4 silenced HBE cells after RSV infection under treatment with AG1478 and U1026, respectively. A. Immunofluorescence analyzed the localization and expressions of c-Jun (n = 10). Scale bar, 100 μm. B. Western blot analysis of c-Jun.

As c-Jun is an important binding site on MUC5AC promoter, we further verify the possible involvement of c-Jun in the overexpression of MUC5AC. The nuclear c-Jun subunit was detected upon stimulation with AG1478 or U0126. Immunofluorescence results demonstrated the nuclear translocation of c-Jun in ITGB4 silenced HBE cells following RSV infection (Figure 6A). As expected, treatments with AG1478, U0126 reduced the nuclear translocation of c-Jun in ITGB4 silenced HBE cells after RSV infection (Figure 6B).

### Blockade of EGFR/ERK1/2/c-Jun pathway inhibited MUC5AC overproduction in ITGB4 silenced HBE cells after RSV infection

**Figure 7:**
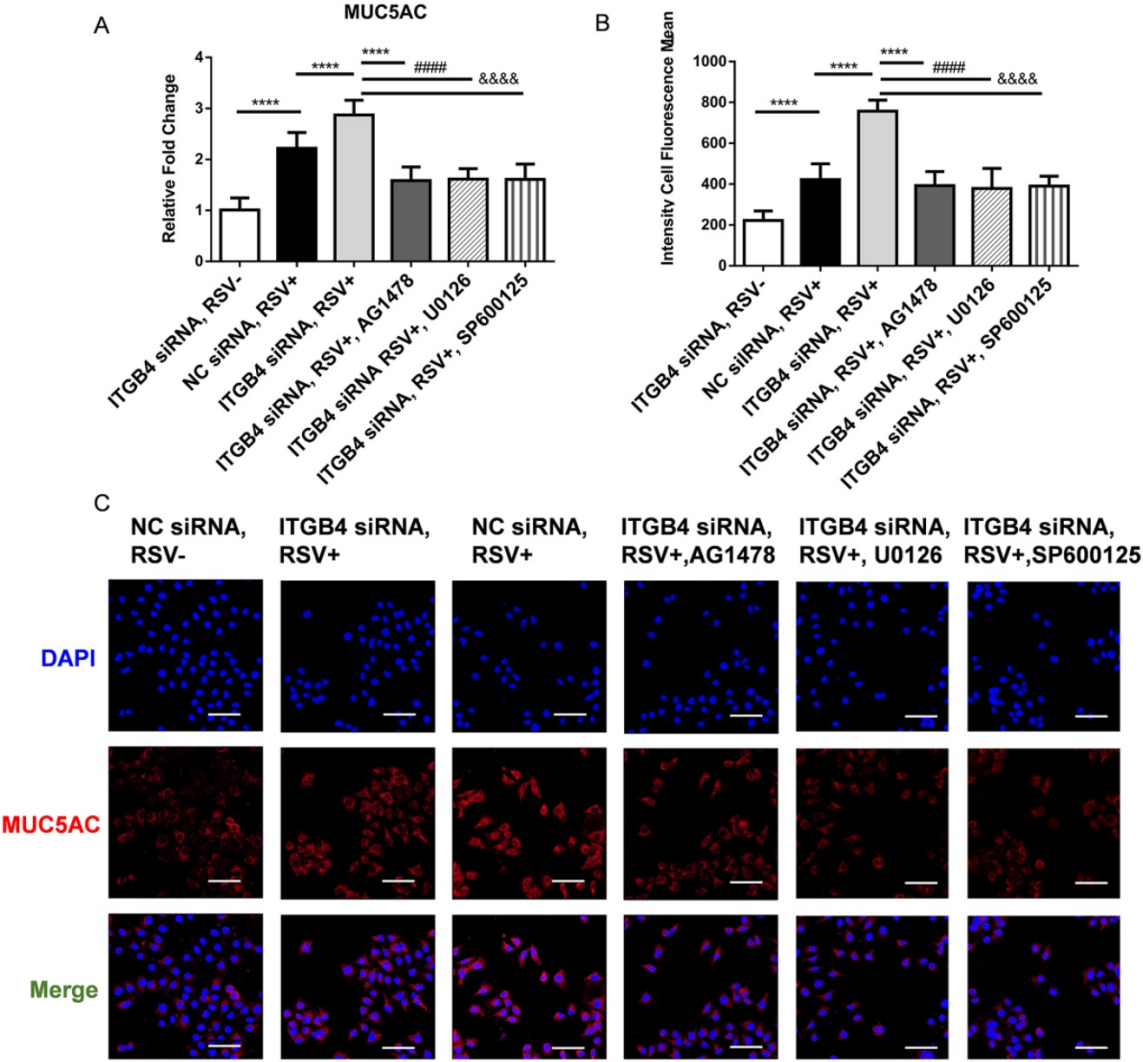
Block of EGFR phosphorylation, ERK1/2 phosphorylation and c-Jun phosphorylation inhibited MUC5AC overexpression in ITGB4 silenced HBE cells after RSV infection. A. qPCR analysis of MUC5AC mRNA expression. B. Level of MUC5AC was assessed by immunofluorescence and quantified by the fluorescence intensity (n = 10). Scale bar, 50 μm. Values are represented as mean ± SEM of at least three independent experiments. **** p<0.001.

To further verify the involvement of EGFR-ERK1/2-c-Jun pathway in the regulation of MUC5AC expression, we detected the level of MUC5AC in ITGB4 silenced HBE cells after RSV infection using AG1478, U0126, SP600125, respectively. Administration of AG1478, U0126 and SP600125 significantly blocked the phosphorylation of EGFR, ERK, c-Jun in ITGB4 silenced HBE cells (Figure 5A, B, C). More importantly, these inhibitor treatments blocked MUC5AC overexpression in ITGB4 silenced HBE cells after RSV infection (Figure 7A, B). These findings verified that ITGB4 deficiency upregulate MUC5AC expression through activation of EGFR/ERK/c-Jun pathway in RSV-infected HBE cells.

### Inhibition of EGFR phosphorylation reduced mucus hypersecretion and MUC5AC overexpression in ITGB4^-/-^ mice after RSV infection

**Figure 8:**
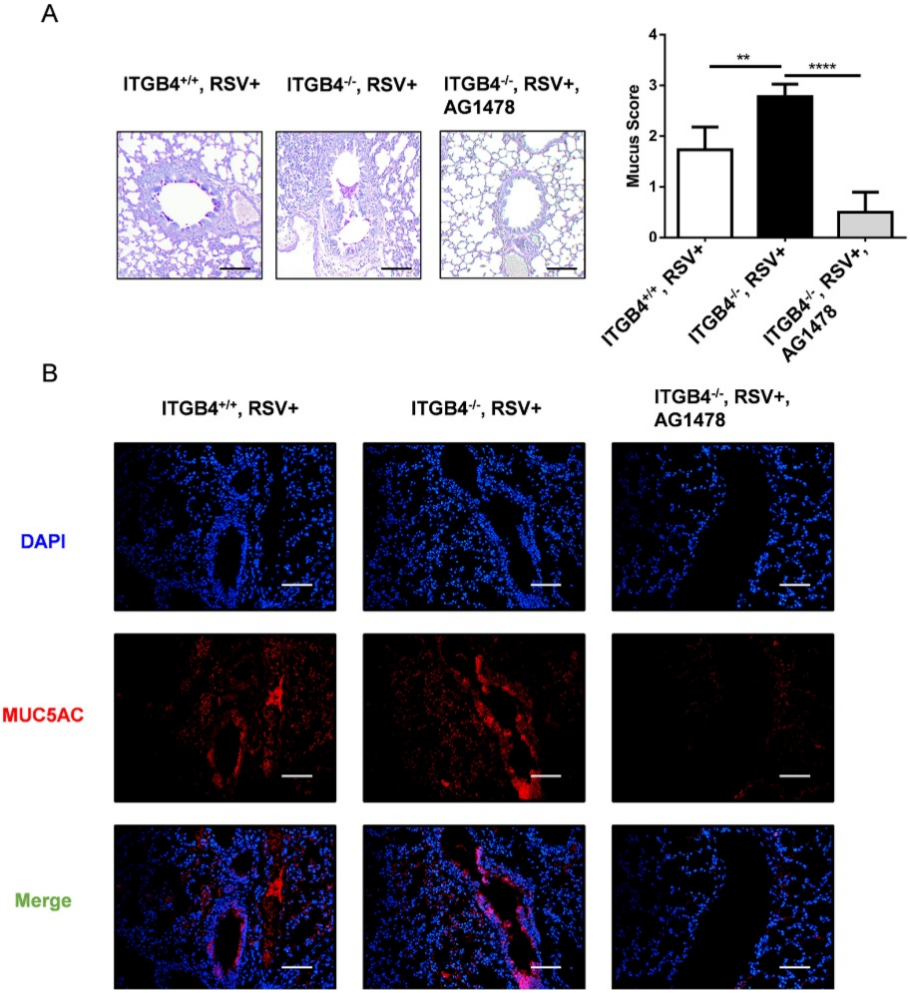
Inhibition of EGFR phosphorylation reduced mucus hypersecretion and MUC5AC overexpression in ITGB4^-/-^ mice after RSV infection. A. Mucus secretion were assessed by PAS staining. B. Level of MUC5AC were assessed by immunofluorescence (n = 10). Scale bar, 25 μm. ** p<0.05; *** p<0.01; **** p<0.001.

As the key role for EGFR is indicated in the pathogenesis of mucus secretion, we block EGFR phosphorylation with AG1478 in ITGB4^-/-^ mice after RSV infection. On day 6 post RSV infection, treatment with AG1478 significantly decreased both mucus hypersecretion in airway lumen and MUC5AC overexpression in airway epithelia (Figure 8A, B). These results verified that EGFR signaling pathway underpins ITGB4-regulated MUC5AC expression in AECs which further contributes to mucus hypersecretion after RSV infection.

## Discussion

Airway mucus is the first biological hydrogel barrier of airway to defend against outside insults. Mucin, as a critical component of mucus, plays an important role in innate immunity which goes beyond the scope of mucociliary. MUC5AC and MUC5B are the principal gel secretory mucins which are important hallmarks of airway pathological state. MUC5B mainly exerts a role in ensuring normal mucus clearance, whereas MUC5AC is the main up-regulated mucin which was involved in the development of AHR, mucous metaplasia, and airway mucus plugging. Moreover, overexpression of MUC5AC is also accompanied with the inducement of airway inflammation 7, 23, 24. It is well known that the expression of MUC5AC in AECs increased obviously in allergic asthma patients 25. Animal models further demonstrated that MUC5AC deficiency enhanced mucociliary transport and antibacterial defense in AECs 26. Meanwhile, mucus plugs were significantly reduced in MUC5AC-deficient mice after allergen stimulation 27. In addition, MUC5AC overexpression in AECs could also imply airway goblet cell metaplasia, which is a key feature of airway remodeling in asthma 28, 29.

Notably, RSV infection is the major trigger for airway immune response and mucus production, which further led to airway obstruction, AHR and airway inflammation. A large body of evidence from epidemiologic studies have shown that RSV infection in infancy and early childhood was associated with elevated susceptibility of later asthma 30. Early RSV infection may have sustained effects on subsequent local lung immune responses 31. Moreover, RSV infection and asthma may share a common host genetic background 30, 32. Intriguingly, there is an obvious heterogeneity in the severity of RSV infection, which may be related to the susceptibility of subsequent asthma. However, the intrinsic mechanism underlying the heterogeneity of RSV infection is far from unclear.

Our previous work found that ITGB4 deficiency in AECs was involved in the development of Th2 responses of allergic asthma though ITGB4-mediated signaling events 13, 33. In this present study, we further pointed out that the decreased expression of ITGB4 was negatively correlated with the level of MUC5AC in throat swab of children with RSV infection. Moreover, ITGB4 deficiency enhances mucus hypersecretion and MUC5AC overexpression following RSV infection in mice model. Furthermore, our findings identify the underpinning molecular mechanisms of mucus hypersecretion in ITGB4 deficient AECs after RSV infection and highlight the potential of targeting this pathway for the treatment of mucus hypersecretion.

Studies have reported that EGFR pathway is necessary for mucus production in AECs 34, 35. The binding of EGF and EGFR in epithelial cells will be triggered after subjection to external stimuli. Activation of EGFR induced receptor dimerization and intracellular domain phosphorylation, which can further activate downstream regulators, including c-Jun N-terminal kinase (JNK), AKT, NF-κB. Of note, the expression of MUC5AC is associated with the activation of c-Jun, AKT and NF-κB pathway 36. Besides, EGFR tyrosine kinase inhibition has also been shown to block the expression of MUC5AC. It has confirmed that EGFR phosphorylation was regulated by ITGB4 in gastric cancer, breast cancer and liver cancer 37. In this study, we further demonstrated that ITGB4 deficiency in AECs induces overproduction of MUC5AC through EGFR activation, and use of EGFR phosphorylation inhibitors could restore expression of MUC5AC significantly 20-22. EGFR is a kind of receptor tyrosine kinase with intrinsic tyrosine kinase activity, which can induce phosphorylation of multiple intracellular substrates, of which ERK1/2 is the most widely known downstream pathway 38. ERK1/2 is a central component in the MAPK decades. After activated by phosphorylated EGFR, ERK1/2 enters the nucleus and acts on transcription factors such as NF-κB and AP-1 39, 40. AP-1, as a type of transcription factor, is composed by subunit c-Jun and subunit c-Fos, which is involved in basal transcription of MUC5AC 41. Our results also proved that ITGB4 and EGER have a direct interaction in human AECs, which is in line with previous findings 11, 22.

Intriguingly, our data also found that ITGB4 deficiency in AECs increased the phosphorylation of EGFR slightly even without RSV infection. However, the level of MUC5AC was enhanced in ITGB4 deficient AECs only under RSV infection. This phenomenon suggests that the expression of MUC5AC was regulated mainly under specific stimulus, such as RSV. Moreover, apart from the ITGB4-EGFR pathways, other components pathways may also participate in the regulation of MUC5AC in AECs 42-44. Not only that, Lakshmanan *et al.* have discovered that MUC5AC interacts directly with ITGB4 in genetically engineered mouse adenocarcinoma tissues 45. However, such direct interaction between ITGB4 and MUC5AC was not observed in our primary AECs which may due to the distinct cellular functions and regulation mechanism of AECs.

In summary, this study found that decreased expression of ITGB4 in AECs was involved in mucus hypersecretion and MUC5AC overexpression through EGFR, ERK and c-Jun pathway following RSV infection. This could serve to better understand the mechanism of ITGB4 deficiency on mucus hypersecretion in RSV-infected AECs. Complete elucidation of the underlying mechanism might provide novel therapeutic targets to reduce mucus overproduction in RSV-related LRTI.

## Supplementary Material

Supplementary figure and tables.

## Abbreviations

RSV: Respiratory syncytial virus
AECs: airway epithelial cells
ITGB4: Integrin β4
AHR: airway hyperresponsiveness
HDM: House Dust Mite

## References

[1] Shi T, McAllister DA, O'Brien KL, Simoes EAF, Madhi SA, Gessner BD (2017). Global, regional, and national disease burden estimates of acute lower respiratory infections due to respiratory syncytial virus in young children in 2015: a systematic review and modelling study. Lancet.

[2] Peebles RS Jr, Graham BS (2005). Pathogenesis of respiratory syncytial virus infection in the murine model. Proc Am Thorac Soc.

[3] Lugo RA, Nahata MC (1993). Pathogenesis and treatment of bronchiolitis. Clin Pharm.

[4] Yu H, Li Q, Kolosov VP, Perelman JM, Zhou X (2012). Regulation of particulate matter-induced mucin secretion by transient receptor potential vanilloid 1 receptors. Inflammation.

[5] Rose MC, Voynow JA (2006). Respiratory tract mucin genes and mucin glycoproteins in health and disease. Physiol Rev.

[6] Ma J, Rubin BK, Voynow JA (2018). Mucins, Mucus, and Goblet Cells. Chest.

[7] Johnson L, Shah I, Loh AX, Vinall LE, Teixeira AS, Rousseau K (2013). MUC5AC and inflammatory mediators associated with respiratory outcomes in the British 1946 birth cohort. Respirology.

[8] Ha EV, Rogers DF (2016). Novel Therapies to Inhibit Mucus Synthesis and Secretion in Airway Hypersecretory Diseases. Pharmacology.

[9] McAllister CS, Ansaldi D, Growcott EJ, Zhong Y, Quackenbush D, Wolff KC (2020). Dexamethasone inhibits respiratory syncytial virus-driven mucus production while increasing viral replication without altering antiviral interferon signaling. Virology.

[10] Shaw LM, Rabinovitz I, Wang HH, Toker A, Mercurio AM (1997). Activation of phosphoinositide 3-OH kinase by the alpha6beta4 integrin promotes carcinoma invasion. Cell.

[11] Chen W, Garcia JG, Jacobson JR (2010). Integrin beta4 attenuates SHP-2 and MAPK signaling and reduces human lung endothelial inflammatory responses. Journal of cellular biochemistry.

[12] Nikolopoulos SN, Blaikie P, Yoshioka T, Guo W, Giancotti FG (2004). Integrin beta4 signaling promotes tumor angiogenesis. Cancer cell.

[13] Xiang Y, Zhou XY, Tan YR, Tan ML, Liu HJ, Liu C (2014). Analysis on the relevance of asthma susceptibility with the alteration of integrin beta 4 expression. PLoS One.

[14] Tang S, Du XZ, Yuan L, Xiao GL, Wu MP, Wang LY (2020). Airway epithelial ITGB4 deficiency in early life mediates pulmonary spontaneous inflammation and enhanced allergic immune response. Journal of Cellular and Molecular Medicine.

[15] Liu C, Yuan L, Zou Y, Yang M, Chen Y, Qu X (2018). ITGB4 is essential for containing HDM-induced airway inflammation and airway hyperresponsiveness. Journal of leukocyte biology.

[16] Kosanovich JL, Eichinger KM, Lipp MA, Yondola MA, Perkins TN, Empey KM (2020). Formulation of the prefusion RSV F protein with a Th1/Th2-balanced adjuvant provides complete protection without Th2-skewed immunity in RSV-experienced young mice. Vaccine.

[17] Wang R, Jiao H, Zhao J, Wang X, Lin H (2018). L-Arginine Enhances Protein Synthesis by Phosphorylating mTOR (Thr 2446) in a Nitric Oxide-Dependent Manner in C2C12 Cells. Oxid Med Cell Longev.

[18] Jiang R, Cai J, Zhu Z, Chen D, Wang J, Wang Q (2014). Hypoxic trophoblast HMGB1 induces endothelial cell hyperpermeability via the TRL-4/caveolin-1 pathway. Journal of immunology.

[19] Yuan L, Zhang X, Yang M, Du X, Wang L, Wu S (2020). Airway epithelial integrin beta4 suppresses allergic inflammation by decreasing CCL17 production. Clin Sci (Lond).

[20] Huafeng J, Deqing Z, Yong D, Yulian Z, Ailing H (2018). A cross-talk between integrin beta4 and epidermal growth factor receptor induces gefitinib chemoresistance to gastric cancer. Cancer cell international.

[21] Bon G, Folgiero V, Di Carlo S, Sacchi A, Falcioni R (2007). Involvement of alpha6beta4 integrin in the mechanisms that regulate breast cancer progression. Breast cancer research: BCR.

[22] Leng C, Zhang ZG, Chen WX, Luo HP, Song J, Dong W (2016). An integrin beta4-EGFR unit promotes hepatocellular carcinoma lung metastases by enhancing anchorage independence through activation of FAK-AKT pathway. Cancer letters.

[23] Groneberg DA, Eynott PR, Oates T, Lim S, Wu R, Carlstedt I (2002). Expression of MUC5AC and MUC5B mucins in normal and cystic fibrosis lung. Respir Med.

[24] Jeong JY, Kim J, Kim B, Kim J, Shin Y, Kim J (2016). IL-1ra Secreted by ATP-Induced P2Y2 Negatively Regulates MUC5AC Overproduction via PLCbeta3 during Airway Inflammation. Mediators Inflamm.

[25] Evans CM, Kim K, Tuvim MJ, Dickey BF (2009). Mucus hypersecretion in asthma: causes and effects. Curr Opin Pulm Med.

[26] Evans CM, Raclawska DS, Ttofali F, Liptzin DR, Fletcher AA, Harper DN (2015). The polymeric mucin Muc5ac is required for allergic airway hyperreactivity. Nat Commun.

[27] Denneny E, Sahota J, Beatson R, Thornton D, Burchell J, Porter J (2020). Mucins and their receptors in chronic lung disease. Clin Transl Immunology.

[28] Whitsett JA (2018). Airway Epithelial Differentiation and Mucociliary Clearance. Ann Am Thorac Soc.

[29] Kim KC (2012). Role of epithelial mucins during airway infection. Pulm Pharmacol Ther.

[30] Fauroux B, Simoes EAF, Checchia PA, Paes B, Figueras-Aloy J, Manzoni P (2017). The Burden and Long-term Respiratory Morbidity Associated with Respiratory Syncytial Virus Infection in Early Childhood. Infect Dis Ther.

[31] Priante E, Cavicchiolo ME, Baraldi E (2018). RSV infection and respiratory sequelae. Minerva Pediatr.

[32] Coutts J, Fullarton J, Morris C, Grubb E, Buchan S, Rodgers-Gray B (2020). Association between respiratory syncytial virus hospitalization in infancy and childhood asthma. Pediatr Pulmonol.

[33] Liu C, Xiang Y, Liu H, Li Y, Tan Y, Zhu X (2010). Integrin beta4 was downregulated on the airway epithelia of asthma patients. Acta Biochim Biophys Sin.

[34] Yu Q, Chen X, Fang X, Chen Q, Hu C (2015). Caveolin-1 aggravates cigarette smoke extract-induced MUC5AC secretion in human airway epithelial cells. Int J Mol Med.

[35] Lv S, Dai C, Liu Y, Sun B, Shi R, Han M (2015). Cell surface protein C23 affects EGF-EGFR induced activation of ERK and PI3K-AKT pathways. J Mol Neurosci.

[36] Le Cras TD, Acciani TH, Mushaben EM, Kramer EL, Pastura PA, Hardie WD (2011). Epithelial EGF receptor signaling mediates airway hyperreactivity and remodeling in a mouse model of chronic asthma. Am J Physiol Lung Cell Mol Physiol.

[37] Takeyama K, Dabbagh K, Lee HM, Agusti C, Lausier JA, Ueki IF (1999). Epidermal growth factor system regulates mucin production in airways. Proc Natl Acad Sci U S A.

[38] Walsh CJ, Zaihra T, Benedetti A, Fugere C, Olivenstein R, Lemiere C (2016). Exacerbation risk in severe asthma is stratified by inflammatory phenotype using longitudinal measures of sputum eosinophils. Clinical and experimental allergy: journal of the British Society for Allergy and Clinical Immunology.

[39] Tsai TF, Lin JF, Lin YC, Chou KY, Chen HE, Ho CY (2019). Cisplatin contributes to programmed death-ligand 1 expression in bladder cancer through ERK1/2-AP-1 signaling pathway. Biosci Rep.

[40] Li X, Bao C, Ma Z, Xu B, Ying X, Liu X (2018). Perfluorooctanoic acid stimulates ovarian cancer cell migration, invasion via ERK/NF-kappaB/MMP-2/-9 pathway. Toxicol Lett.

[41] Meng S, Wu Y, Hu X, Zhang H, Li C (2015). [Naringenin may block RSV-induced mucous hypersecretion in A549 cell via JNK/AP-1 signaling pathway]. Zhonghua Er Ke Za Zhi.

[42] Xu Q, Chen LX, Ran DH, Xie WY, Li Q, Zhou XD (2017). Bombesin receptor-activated protein regulates neutrophil elastase-induced mucin5AC hypersecretion in human bronchial epithelial cells. Exp Cell Res.

[43] Wu S, Li H, Yu L, Wang N, Li X, Chen W (2017). IL-1beta upregulates Muc5ac expression via NF-kappaB-induced HIF-1alpha in asthma. Immunol Lett.

[44] Hewson CA, Haas JJ, Bartlett NW, Message SD, Laza-Stanca V, Kebadze T (2010). Rhinovirus induces MUC5AC in a human infection model and in vitro via NF-kappaB and EGFR pathways. Eur Respir J.

[45] Lakshmanan I, Rachagani S, Hauke R, Krishn SR, Paknikar S, Seshacharyulu P (2016). MUC5AC interactions with integrin beta4 enhances the migration of lung cancer cells through FAK signaling. Oncogene.

