# Supplementary material for: ITGB4 deficiency induces mucus hypersecretion by upregulating MUC5AC in RSV-infected airway epithelial cells
Source: Int J Biol Sci. 2022 Jan 1;18(1):349–59. doi: 10.7150/ijbs.66215 (PMC8692133; doi:10.7150/ijbs.66215)

Table S1. Primer sequence of genes for PCR and qPCR.

Gene	Primer sequence
ITGB4 (human)	Forward: 5'- CACCTCCGTCTCCTCCCAC -3'
	Reverse: 5'- GTTGGGGATGTTGAGCCGAT -3'
MUC5AC (human)	Forward: 5'- AGCCGGGAACCTACTACTCG -3'
	Reverse: 5'- AAGTGGTCATAGGCTTCGTGC -3'
β-actin (human)	Forward: 5'- TTGCAGCTCCTTCGTTGCC -3'
	Reverse: 5'- GACCCATTCCCACCATCACA -3'
MUC5AC (mouse)	Forward: 5'- GTGGTTTGACACTGACTTCCC -3'
	Reverse: 5'- CTCCTCTCGGTGACAGAGTCT -3'
β-actin (mouse)	Forward: 5'- ATATCGCTGCGCTGGTCGTC -3'
	Reverse: 5'- AGGATGGCGTGAGGGAGAGC-3'

Table S2. Demographic characteristics of RSV patients and healthy controls.

	Healthy controls	RSV patients
Number of subjects	15	11
Sex(F/M)	7/8	6/5
Age	4.40[0.08-11]	2.91[0.25-4]
<24 months	10	8
Only RSV	0	8
RSV+ADV	0	0
RSV+IFV	0	1
RSV+PIV	0	0
RSV+UU	0	2
WBC (10^9/L)	7.33 [4.5-12.1]	10.40 [6.5-14.3] ***
CRP (mg/L)	2.65[0.02-6.83]	13[5.0-21.17] ***

All data are presented as median. ADV = adenovirus, PIV = parainfluenza virus, IFV= influenza virus, UU = mycoplasma, WBC = whole blood cell count, CRP = C-reactive protein. Controls VS RSV patients (Unpaired t test). *** $p<0.01$; **** $p<0.001$

Figure S1. Immunofluorescence staining of MUC5AC and ITGB4 in ITGB4$^{-/-}$ mice after RSV infection. (n = 10). Scale bar, 25 μm.

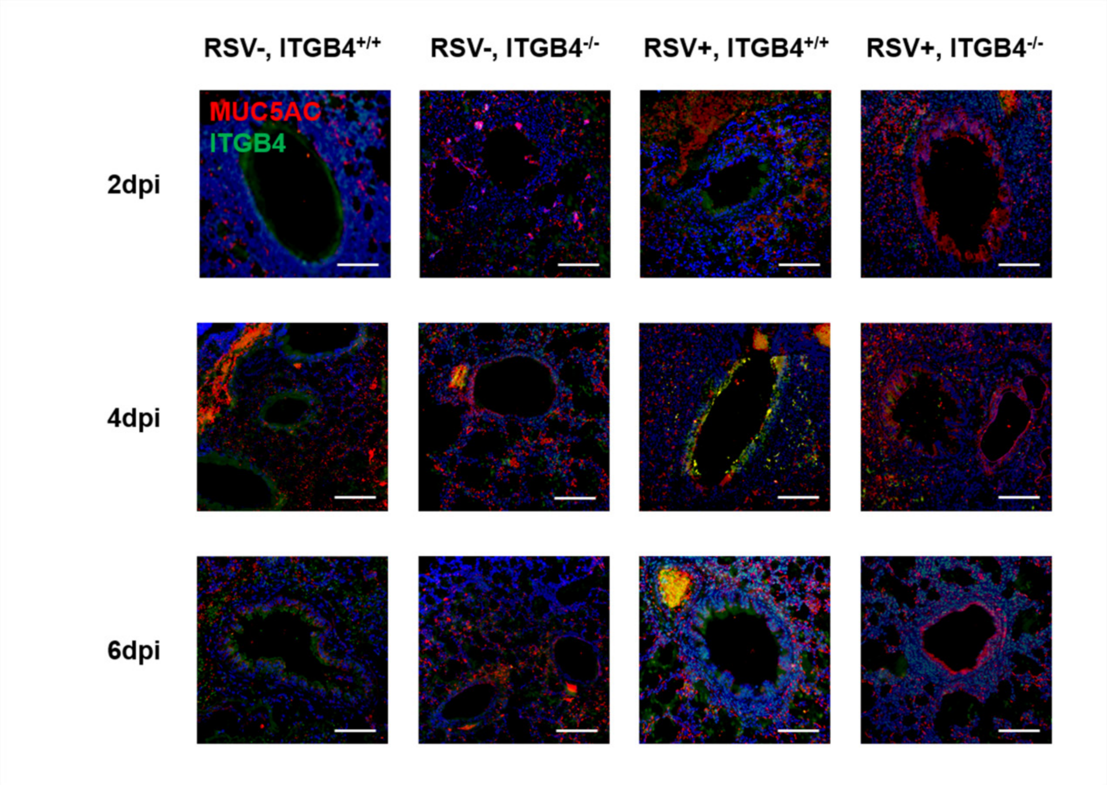

Supplement: Supplementary file 1 — Supplementary figure and tables. [file ijbsv18p0349s1.pdf]
